# Consequences of the Expanding Global Distribution of *Aedes albopictus* for Dengue Virus Transmission

**DOI:** 10.1371/journal.pntd.0000646

**Published:** 2010-05-25

**Authors:** Louis Lambrechts, Thomas W. Scott, Duane J. Gubler

**Affiliations:** 1 Department of Virology, Institut Pasteur, Paris, France; 2 Department of Entomology, University of California, Davis, United States of America; 3 Duke University-National University of Singapore Graduate Medical School, Singapore; 4 Asia-Pacific Institute of Tropical Medicine and Infectious Diseases, University of Hawaii, Honolulu, United States of America; Pediatric Dengue Vaccine Initiative, United States of America

## Abstract

The dramatic global expansion of *Aedes albopictus* in the last three decades has increased public health concern because it is a potential vector of numerous arthropod-borne viruses (arboviruses), including the most prevalent arboviral pathogen of humans, dengue virus (DENV). *Ae. aegypti* is considered the primary DENV vector and has repeatedly been incriminated as a driving force in dengue's worldwide emergence. What remains unresolved is the extent to which *Ae. albopictus* contributes to DENV transmission and whether an improved understanding of its vector status would enhance dengue surveillance and prevention. To assess the relative public health importance of *Ae. albopictus* for dengue, we carried out two complementary analyses. We reviewed its role in past dengue epidemics and compared its DENV vector competence with that of *Ae. aegypti*. Observations from “natural experiments” indicate that, despite seemingly favorable conditions, places where *Ae. albopictus* predominates over *Ae. aegypti* have never experienced a typical explosive dengue epidemic with severe cases of the disease. Results from a meta-analysis of experimental laboratory studies reveal that although *Ae. albopictus* is overall more susceptible to DENV midgut infection, rates of virus dissemination from the midgut to other tissues are significantly lower in *Ae. albopictus* than in *Ae. aegypti*. For both indices of vector competence, a few generations of mosquito colonization appear to result in a relative increase of *Ae. albopictus* susceptibility, which may have been a confounding factor in the literature. Our results lead to the conclusion that *Ae. albopictus* plays a relatively minor role compared to *Ae. aegypti* in DENV transmission, at least in part due to differences in host preferences and reduced vector competence. Recent examples of rapid arboviral adaptation to alternative mosquito vectors, however, call for cautious extrapolation of our conclusion. Vector status is a dynamic process that in the future could change in epidemiologically important ways.

## Introduction

The past three decades have seen a dramatic global expansion in the geographic distribution of *Aedes* (*Stegomyia*) *albopictus* (Skuse) that continues today [1]. This has caused considerable concern among some scientists and public health officials over the possibility that range expansion by this species will increase the risk of arthropod-borne virus (arbovirus) transmission [2], [3]. Since 2004, this concern has been amplified by the implication of *Ae. albopictus* in chikungunya outbreaks on islands in the Indian Ocean and in central Africa and Italy [4]–[6]. The possibility of *Ae. albopictus* changing the transmission dynamics of both introduced and indigenous arboviral diseases, and increasing the risk of human infection, has stimulated increased vectorial capacity research on this species in the past two decades. *Ae. albopictus* appears to be susceptible to infection with, and is able to transmit, most viruses for which it has been experimentally tested, including eight alphaviruses, eight flaviviruses, and four bunyaviruses, representing the three main arbovirus genera that include human pathogens (reviewed in [7]).

In addition to chikungunya virus, the only other human pathogens known to be transmitted in epidemic form by *Ae. albopictus* are the four serotypes of dengue virus (DENV-1, -2, -3, and -4). Dengue is the most prevalent human arboviral infection worldwide. *Ae. albopictus* was reportedly responsible for dengue epidemics in Japan and Taipei, Taiwan during World War II [8]. More recently, it was associated with dengue epidemics in the Seychelles Islands (1977), La Réunion Island (1977), China (1978), the Maldive Islands (1981), Macao (2001), and Hawaii (2001) ([9]–[12]; D. Fontenille, personal communication; D. J. Gubler, unpublished data). The few dengue epidemics attributed to *Ae. albopictus*, however, were essentially classical dengue fever. Although a few severe and fatal cases of hemorrhagic disease may have occurred, these were not typical dengue hemorrhagic fever epidemics. In fact, all major epidemics of dengue hemorrhagic fever have occurred only in areas where *Ae. aegypti* is found. During the past three decades this species, which is closely related to *Ae. albopictus*, was considered the principal vector in the global resurgence of epidemic dengue [13], [14]. In this article, we attempt to clarify the public health consequences of range expansion by *Ae. albopictus* by assessing its importance to DENV transmission relative to *Ae. aegypti*. We used two complementary approaches: (i) examination of dengue incidence records in places where *Ae. albopictus* was present in the absence of *Ae. aegypti* (“natural experiments”) and (ii) meta-analysis of published experimental studies on the relative vector competence of both species for DENV.

## Historical Background

*Ae. albopictus* is a day-biting species that belongs to the subgenus *Stegomyia* [15]. Originally a zoophilic forest species from Asia, *Ae. albopictus* spread west to islands in the Indian Ocean and east to islands in the Pacific Ocean in the 19th and first half of the 20th century [16]. During the subsequent 30 years there was no reported movement of this species to new areas. In the 1980s, however, *Ae. albopictus* began a dramatic geographic expansion that continues to the present day [1]. It was first reported in Albania in 1979 [17], Texas in 1985 [18], and Brazil in 1986 [19]. In the following two decades, *Ae. albopictus* became established in many countries in the Americas ranging from the US to Argentina, in at least four countries in Central Africa (Nigeria, Cameroon, Equatorial Guinea, and Gabon), 12 countries in Europe (Albania, Bosnia and Herzegovina, Croatia, Greece, France, Italy, Montenegro, The Netherlands, Serbia, Slovenia, Spain, and Switzerland), several islands in the Pacific and the Indian Oceans, and Australia (reviewed in [2], [7]). Introductions were documented in several other countries (e.g., New Zealand, Barbados, Trinidad) where it was eliminated or did not become established. This rapid spread in geographic range around the world was most likely the result of changes in the shipping and used tire industries [20].

*Ae. albopictus* is a generalist that readily adapts to diverse environmental conditions in both tropical and temperate regions [21]. Like *Ae. aegypti*, it is adapted to the peridomestic environment where it feeds on humans and domestic animals and oviposits in a variety of natural and artificial water holding containers [22]. In the 18th and 19th centuries, it was the dominant day-biting species in most Asian cities [23]. As the shipping industry expanded, *Ae. aegypti* gradually replaced *Ae. albopictus* as the dominant day-biting mosquito in Asian cities because it was better adapted to the urban environment [24]. By the middle of the 20th century, both species were found in most cities in Asia, but *Ae. albopictus* was relegated to gardens with tropical vegetation [23]. In some island communities of the Pacific, however, the reverse occurred. *Ae. aegypti* never became established in northern Taiwan, and was eliminated from Guam, Saipan, and the islands of Hawaii by a combination of intense control directed at urban habitats and competition from *Ae. albopictus* in the more densely vegetated peridomestic habitat.

## Natural Experiments

Three locations (Taipei, Guam, and Hawaii) provide meaningful case studies on the relative potential of *Ae. albopictus* and *Ae. aegypti* as epidemic DENV vectors. *Ae. albopictus* was the dominant or only day-biting *Stegomyia* species on these three islands for over 50 years, a period when epidemic dengue expanded geographically and greatly increased in frequency in the Pacific Basin. If *Ae. albopictus* was an efficient epidemic DENV vector, one would have expected numerous dengue epidemics in places where it predominated when epidemics were occurring on nearby islands or areas infested with *Ae. aegypti*. Although comprehensive data were not always available to establish the relative contribution of *Ae. aegypti* and/or *Ae. albopictus* to DENV transmission, the fact that there were no major dengue epidemics on Guam or Hawaii, nor in those areas where *Ae. aegypti* is not sympatric to *Ae. albopictus* on Taiwan, is consistent with speculation [25] that *Ae. albopictus* is not an efficient epidemic DENV vector.

### Taiwan

*Ae. aegypti* has infested the southern third of Taiwan since the 19th century, but never became established in the metropolitan area of Taipei in the northern part of the island (J. C. Lien, personal communication). During the Japanese occupation of Taiwan, *Ae. albopictus* population densities were high because of the large number of water storage tanks kept for firefighting (J. C. Lien, personal communication). After World War II, indoor spraying of DDT during the malaria eradication program helped to eliminate *Ae. aegypti* from all but the most southern tip of the island. *Ae. albopictus* occurs naturally throughout Taiwan and its distribution was not known to be affected by the malaria eradication program, perhaps because it preferred sylvan habitats to human habitations. Taiwan was free of epidemic DENV transmission from 1945 until 1981; i.e., about 35 years without disease. In 1981, a DENV-2 epidemic occurred on Liuchiu Island, off the southern tip of Taiwan, where *Ae. aegypti* was common ([26], [27]; D. J. Gubler, unpublished data). In 1987–1988, another larger epidemic of DENV-1 occurred in Kaohsiung and other southern cities that had been reinfested by *Ae. aegypti*. From 1989 to 2009, Taiwan reported several dengue outbreaks, some with hemorrhagic disease, and many imported cases. All four DENV serotypes were involved, but most hemorrhagic disease was associated with DENV-2 and DENV-3. Most local transmission occurred in the southern part of the island where *Ae. aegypti* occurred. There were no autochthonous cases reported in other parts of the island where *Ae. albopictus* was the only day-biting *Stegomyia* species until 1995–1996, when sporadic autochthonous dengue cases were reported from Taipei, an area where surveys showed that only *Ae. albopictus* occurred (J. C. Lien, personal communication). In both years, DENV-1 was isolated from *Ae. albopictus* collected in the outbreak area of Taipei, as well as from humans. Although these incidents created concern among health officials, they were expected because many dengue cases were imported each year from southeast Asian countries to the southern part of Taiwan and other areas where *Ae. albopictus* was common. Although at that time Taipei had a dense, crowded human population of about three million people with low herd immunity to all four DENV serotypes and *Ae. albopictus* was common in the city, a major dengue epidemic did not occur.

### Guam and the Northern Mariana Islands

Guam was infested with *Ae. aegypti* during World War II and experienced dengue outbreaks as a part of the Pacific-wide DENV-1 epidemic that occurred from 1941 to 1945. Although it is not known exactly when *Ae. aegypti* was eliminated from Guam, *Ae. albopictus* became the dominant day-biting *Stegomyia* species sometime during the 1960s. Because of the reintroduction of dengue into the Pacific in the 1970s and increased epidemic activity during the past four decades caused by all four serotypes, it seems reasonable to expect that outbreaks would have occurred on Guam and other Mariana Islands, such as Saipan. Dengue epidemics were documented on nearby island groups, Palau in 1988 and 1995 [28], [29] and Yap in 1995 and 2004 [30], [31]. Investigations showed that both Palau and Yap were infested with *Ae. aegypti*, although *Ae. hensilli*, an indigenous member of the *Ae. scutellaris* complex, was shown to be the epidemic vector on Pellilieu, Palau in 1988 and on Europik, Yap in 1995 [29], [31]. Neither Guam nor Saipan have had an epidemic of dengue during the 38 years since dengue was re-introduced to the Pacific islands in 1971, even though *Ae. albopictus* is widespread on both islands.

### Hawaii

Hawaii also experienced a major dengue outbreak in 1943–1944 during the Pacific DENV-1 epidemic. *Ae. aegypti* was eliminated from Oahu in the 1960s, but *Ae. albopictus* remained a common peridomestic mosquito on all of the Hawaiian islands, including Oahu and the Honolulu metropolitan area. There were two reported dengue cases in German tourists in 1995, but they could not be properly documented and were most likely false positives ([32]; D. J. Gubler and A. V. Vorndam, unpublished data). Similarly, a case of febrile illness with positive IgM antibody was reported from Hawaii in 1998. Follow-up, however, showed that it was a false positive laboratory test from a commercial kit (P. Effler, D. Morens, A. V. Vorndam and D. J. Gubler, unpublished data). In 2001–2002, 122 autochthonous dengue cases with no hemorrhagic disease were reported. The causal DENV-1 was imported from French Polynesia [12], [33]. This was the only dengue outbreak that occurred in 56 years in Hawaii, despite thousands of dengue cases that have likely been imported during this period into an area with high population densities of *Ae. albopictus* and low human herd immunity.

## Ecology and Host Preference

In the “natural experiments” examined above, the much lower dengue activity despite low herd immunity in human populations, occurrence of epidemic activity at nearby locations, numerous imported cases, and presence of *Ae. albopictus* as the predominant or only *Stegomyia* species, are consistent with the conclusion that *Ae. albopictus* is a less efficient epidemic dengue vector than *Ae. aegypti*. Usual explanations for this difference are based on different ecologies of the two species. *Ae. aegypti* is well-adapted to the highly urban environments of tropical cities, living in intimate association with humans, while *Ae. albopictus* is better adapted to peridomestic settings with vegetation that provides its preferred larval development and resting sites [23], [34], [35]. Although *Ae. albopictus* is found occasionally to feed and rest inside human dwellings [35]–[37], it is more commonly found outdoors where it has increased contact with other animals and decreased contact with humans. Both species feed readily on humans, but whereas *Ae. aegypti* rarely feeds on other animals, *Ae. albopictus* is a catholic feeder, taking blood from a variety of animal species [38]. This characteristic makes it a potentially dangerous bridge vector of zoonotic pathogens to humans, but conversely is expected to decrease its efficiency as an epidemic vector of pathogens restricted to humans.

Although the opportunistic and zoophilic feeding behavior of *Ae. albopictus* clearly influences its efficiency as an epidemic arbovirus vector, some observations indicate that it might not be the only explanation. Analysis of blood meals in wild mosquitoes [39], [40] and host choice experiments [41] showed that when given the choice, *Ae. albopictus* prefers to bite humans over other animals. Depending on host availability, the almost exclusive anthropophily of *Ae. aegypti* may, therefore, not be sufficient to explain the higher vectorial capacity for DENV of *Ae. aegypti* relative to *Ae. albopictus*. In Thailand, for example, analysis of blood meals revealed a high percentage of human feeding by *Ae. albopictus,* similar to *Ae. aegypti* [42]. At two sites in Southern Thailand, ∼95% of *Ae. albopictus* blood meals were taken exclusively from humans, and all mixed meals included a human. Thus, at least in some areas, vertebrate host associations cannot entirely explain the observed minor role played by *Ae. albopictus* in DENV transmission.

## Oral Susceptibility

Results from studies on the relative susceptibility of *Ae. albopictus* versus *Ae. aegypti* to oral DENV infection have produced conflicting results [43]–[47]. In order to disentangle these inconsistencies, we conducted a meta-analysis of 14 studies published between 1971 and 2009 that compared oral susceptibility of *Ae. albopictus* and *Ae. aegypti* for DENV [43]–[56] (for details see Methods and Supporting Information). Whereas vectorial capacity encompasses all environmental, ecological, behavioral, and molecular factors underlying an insect's role in pathogen transmission, vector competence is a subcomponent of vectorial capacity and is defined as the intrinsic ability of a vector to become infected with, allow replication of, and subsequently transmit a pathogen to a susceptible host [57]. Two major “barriers” in mosquitoes that can prevent or limit viral transmission have been described in the literature, namely a “midgut infection barrier” and a “midgut escape barrier” [58]. A “salivary gland infection barrier” and a “salivary gland escape barrier” have also been suggested but they are controversial in the case of DENV in *Ae. aegypti* and *Ae. albopictus*. Although the exact nature of these barriers remains to be elucidated, they have inspired the definition of vector competence indices based on virus progression through the mosquito: midgut infection, virus dissemination from the midgut (typically measured by detection of viral antigen in head tissues), and virus presence in salivary glands and/or salivary secretions. Of the 91 separate experiments that met our inclusion criteria, 39 estimated vector competence based on the proportion of mosquitoes with a midgut infection, 41 measured the proportion of mosquitoes with a disseminated infection, and 11 experiments measured both. Only one study detected virus in salivary glands and salivary secretions [48] so that these indices could not be meta-analyzed. We examined the two other vector competence indices separately.

### Midgut Infection

**Figure 1:**
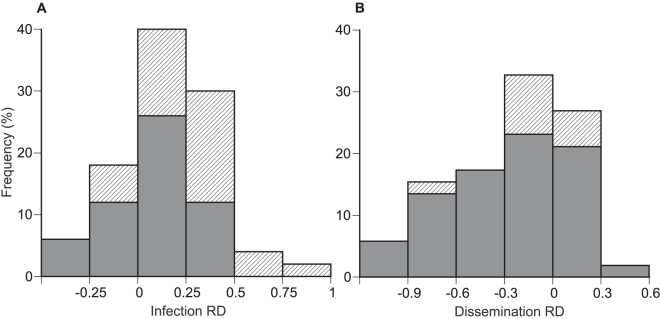
Distribution of RD among published experiments comparing the vector competence of *Ae. albopictus* and *Ae. aegypti* for horizontal transmission of DENV. Graphs show the overall frequency of differences in (A) the proportion of infected mosquitoes and (B) the proportion of mosquitoes with an infection disseminated from the midgut, as a function of the mosquito colonization history (i.e., number of generations spent in the laboratory before vector competence was assessed). Filled bars represent mosquitoes held ≤5 generations in the laboratory; shaded bars correspond to mosquitoes colonized for >5 generations. Negative RD values represent a reduced rate whereas positive values represent a greater rate for *Ae. albopictus* compared to *Ae. aegypti*.

**Figure 2:**
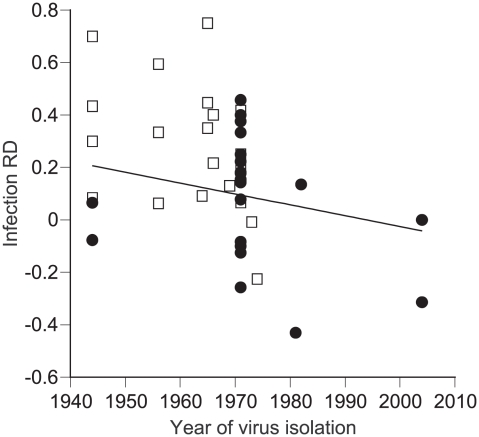
Relationship between RD and virus isolation year among published experiments comparing DENV midgut infection in *Ae. albopictus* and *Ae. aegypti*. Each point represents a single experiment. Different symbols indicate a different mosquito colonization history (i.e., number of generations spent in the laboratory before vector competence was assessed). Filled circles represent mosquitoes held ≤5 generations in the laboratory; open squares correspond to mosquitoes colonized for >5 generations. The solid line shows the linear regression (R^2^ = 0.162, *P* = 0.007). Negative RD values represent a reduced rate whereas positive values represent a greater rate for *Ae. albopictus* compared to *Ae. aegypti*.

Assuming no data structure, cumulative rate difference (RD) across experiments was 16%. The bootstrapped, bias-corrected 95% confidence interval (10%–24%) did not bracket zero, indicating that the effect was statistically significant. Because we had arbitrarily assigned positive values of RD to a greater midgut infection rate for *Ae. albopictus* compared to *Ae. aegypti*, this result showed that, overall, *Ae. albopictus* had a higher midgut susceptibility to DENV infection than *Ae. aegypti*. The total heterogeneity of the data was marginally insignificant when tested against a χ^2^ distribution (Q_T_ = 65.6, d.f. = 49, *P* = 0.057), which was suggestive of underlying data structure. Of the two categorical and four continuous variables that were tested as predictors of RD, only two explained a statistically significant portion of RD heterogeneity. First, mosquito colonization history explained 11% of total heterogeneity (Table 1). Cumulative RD was not statistically different from zero for mosquitoes held fewer than five generations in the laboratory. It was about three times higher and significantly greater than zero for mosquitoes that had been colonized for more than five generations (Table 1). Although *Ae. albopictus* appeared to be, overall, more susceptible to DENV midgut infection than *Ae. aegypti*, this effect was largely due to experiments that used mosquito colonies maintained in the laboratory for many generations (Figure 1). Second, the year of virus isolation explained 13% of the total data heterogeneity (Table 2). Regression of RD as a function of the year of virus isolation indicated that RD decreased with the time elapsed since the virus was isolated. Examination of this regression including mosquito colonization history revealed that the year of virus isolation was likely confounded with the number of generations mosquitoes spent in the laboratory (Figure 2). More recent studies tended to use viruses that were isolated more recently and mosquitoes that were maintained in the laboratory for a short time, probably because of increased awareness of the importance of using specimens representative of natural systems. Although in our analysis dependence of mosquito colonization history and virus isolation year prevents us from drawing a firm conclusion, *Ae. albopictus* vector competence was previously reported to be positively associated with time in colonization [46]. Although the overall effect of different virus serotypes was not statistically significant, RD was significantly greater than zero for DENV-1 and DENV-3, but not different from zero for DENV-2 and DENV-4, suggesting that the susceptibility of *Ae. albopictus* relative to *Ae. aegypti* may vary across serotypes.

**Table 1:** Influence of categorical factors on the relative oral susceptibility to DENV of *Ae. albopictus* compared to *Ae. aegypti* measured by the rate of midgut infection and the rate of virus dissemination from the midgut.

Factor	Class	Infection					Dissemination				
		#Exp	RD	95% C.I.	Q_M_/Q_T_	*P-Value*	#Exp	RD	95% C.I.	Q_M_/Q_T_	*P-Value*
Mosquito colonization	≤5 generations	28	0.080	−0.011 to 0.164	0.109	0.040	43	−0.290	−0.405 to −0.179	0.041	0.122
	>5 generations	22	0.244	0.144 to 0.350			9	−0.103	−0.255 to 0.014		
Serotype	DENV-1	11	0.305	0.161 to 0.462	0.131	0.137	4	−0.318	−0.822 to 0.202	0.067	0.266
	DENV-2	26	0.080	−0.013 to 0.159			44	−0.277	−0.374 to −0.179		
	DENV-3	10	0.183	0.066 to 0.292			2	−0.024	−0.167 to 0.122		
	DENV-4	3	0.179	−0.200 to 0.593			2	0.152	−0.100 to 0.399		

For each class of individual factors the number of experiments (#Exp), mean rate difference (RD) and its bootstrapped, bias-corrected 95% confidence interval (95% C.I.) are indicated. The influence of each factor was characterized using separate one-way mixed-model analyses in *Metawin 2.0* [74]. Effects were quantified by partitioning the total heterogeneity in effect size of the sample (Q_T_) into the heterogeneity explained by the factor (Q_M_) and the residual heterogeneity. A significant *P*-value implies that there are differences in mean effect size among classes.

**Table 2:** Influence of continuous variables on the relative oral susceptibility to DENV of *Ae. albopictus* compared to *Ae. aegypti* measured by the rate of midgut infection and the rate of virus dissemination from the midgut.

Variable	Infection						Dissemination					
	#Exp	Median (range)	RD	95% C.I.	Q_M_/Q_T_	*P-Value*	#Exp	Median (range)	RD	95% C.I.	Q_M_/Q_T_	*P-Value*
Virus isolation year	44	1971 (1944–2004)	0.180	0.10 to 0.263	0.127	0.007	44	1974 (1944–2004)	−0.258	−0.368 to −0.155	0.022	0.397
Passage number	41	2 (1–27)	0.194	0.105 to 0.280	0.019	0.403	33	5 (1–27)	−0.357	−0.500 to −0.216	0.025	0.422
Extrinsic incubation period	50	14 d (7–21)	0.161	0.087 to 0.236	0.023	0.231	52	14 d (12–21)	−0.255	−0.356 to −0.158	0.039	0.181
Sample size	50	31.5 (8–1,289)	0.161	0.090 to 0.237	0.001	0.794	52	63 (21–1,289)	−0.255	−0.344 to −0.160	0.000	0.916

For each individual variable, the number of experiments included in the analysis (#Exp), median value and range, mean rate difference (RD) and its bootstrapped, bias-corrected 95% confidence interval (95% C.I.) are given. The influence of each variable was characterized using separate one-way mixed-model analyses in *Metawin 2.0* [74]. Effects were quantified by partitioning the total heterogeneity in effect size of the sample (Q_T_) into the heterogeneity explained by the regression model (Q_M_) and the residual heterogeneity. A significant *P*-value indicates that the variable explains a significant amount of the variability in effect size.

### Disseminated Infection

Assuming no data structure, cumulative RD across experiments was −26%. The bootstrapped, bias-corrected 95% confidence interval (−36 to −16%) did not bracket zero, indicating that this effect was statistically significant. Negative values of RD indicate a lower rate of virus dissemination for *Ae. albopictus* compared to *Ae. aegypti*, showing that, overall, *Ae. albopictus* was less susceptible to DENV dissemination from the midgut than *Ae. aegypti*. Total heterogeneity of the sample was not significant when tested against a χ^2^ distribution (Q_T_ = 43.9, d.f. = 51, *P* = 0.751), which is consistent with the absence of major data structure. Accordingly, none of the factors analyzed explained a statistically significant portion of RD heterogeneity (Tables 1 and 2). Although the effect was not statistically significant overall, dissemination RD decreased with mosquito colonization history. Cumulative RD was not significantly different from zero for mosquitoes colonized for more than five generations; it was about three times larger and significantly smaller than zero for mosquitoes that had spent fewer than five generations in the laboratory (Figure 1; Table 1). When virus dissemination from the midgut was considered, *Ae. albopictus* was, overall, less susceptible to DENV infection than *Ae. aegypti*. This effect was reduced in experiments that used mosquito colonies maintained in the laboratory for more than a few generations. Although the overall effect of serotype was not statistically significant, RD was significantly smaller than zero for DENV-2, but not different from zero for the three other serotypes. Interpretation of this result in terms of relative susceptibility to different serotypes is difficult because of the over-representation of DENV-2 in the analysis of dissemination (44 experiments out of 52).

Taken together, our meta-analysis indicates that inconsistency when comparing experimental vector competence of *Ae. albopictus* and *Ae. aegypti* for DENV was likely due to two factors. First, the relative difference between both species appeared to differ according to whether vector competence was measured as the proportion of mosquitoes with a midgut infection or as the proportion of mosquitoes with a disseminated infection. Although *Ae. albopictus* was, overall, more susceptible than *Ae. aegypti* to midgut infection, the rate of virus dissemination to other tissues was lower for *Ae. albopictus*. That *Ae. albopictus* displayed, overall, a smaller proportion of individuals with disseminated infections despite including a larger proportion of midgut-infected individuals than *Ae. aegypti* (due to its higher susceptibility to midgut infection) reinforces the conclusion that DENV dissemination is less efficient in *Ae. albopictus* than in *Ae. aegypti*. This result across a broad range of studies confirms the observation made in a recent report that examined both vector competence indices [45]. Second, the relative difference between *Ae. albopictus* and *Ae. aegypti* for both indices increased with the number of generations experimental mosquitoes spent in the laboratory. In other words, the susceptibility of *Ae. albopictus* for DENV appears to increase with time in colonization whereas it is not the case, or to a smaller extent, for *Ae. aegypti*. This latter result emphasizes the importance of using fresh material, recently derived from the field, to reach meaningful conclusions.

A complicating factor between the two species may be related to the endosymbiotic bacteria *Wolbachia*, which naturally infects *Ae. albopictus* [59],[60] and is absent in wild *Ae. aegypti* [61], [62]. *Wolbachia* infection has been shown to protect insects against viral infections [63] and may be lost accidentally during lab colonization, perhaps by inclusion of antibiotics in laboratory diets, effect of larval crowding [64], or increased larval rearing temperatures [64], [65]. Accidental loss or attenuation of *Wolbachia* infection could result in loss of *Ae. albopictus* antiviral protection. This hypothesis needs to be tested.

Our meta-analysis indicates that despite its relatively higher susceptibility to midgut infection compared to *Ae. aegypti*, the lower rate of virus dissemination is likely an important factor in the minor role of *Ae. albopictus* as an epidemic vector of DENV. Although this conclusion is based on experimental assessments of vector competence in the laboratory, the broad variety of experimental settings included in the meta-analysis indicates that the overall effect did not result from conditions specific to a particular experiment.

## Vertical Transmission

Our conclusion that DENV dissemination rate is lower in *Ae. albopictus* than in *Ae. aegypti* raises questions about the relative rate of DENV vertical transmission in both species and its impact on natural DENV maintenance cycles [66]. Unfortunately, the very limited number of comparative studies available on the topic did not allow us to perform a meta-analysis. Of three studies that compared rates of DENV vertical transmission experimentally in *Ae. albopictus* and *Ae. aegypti*, two reported that vertical transmission was more efficient in *Ae. albopictus* [67], [68] and one suggested otherwise [69]. In the earliest study, despite substantial variation between virus strains and serotypes, experimental rates of vertical transmission of all four DENV serotypes were much higher in *Ae. albopictus* than in *Ae. aegypti* [68]. This study, however, used mosquito colonies that were maintained for many generations in the laboratory, which might have biased the outcome of the experiments as was observed in our meta-analysis of oral susceptibility. Moreover, in that study mosquitoes were infected by intrathoracic (IT) inoculation, so that both midgut infection and midgut escape barriers were bypassed. If low rates of virus dissemination in *Ae. albopictus* were due to an efficient midgut escape barrier, it would not be expected to play an important role in IT-inoculated mosquitoes.

In a different study, vertical transmission rates for DENV-1 (i.e., percentage of females producing infected offspring) ranged from 11% to 41% and filial infection rate (i.e., percentage of offspring infected) ranged from 0.5% to 3% among multiple geographical strains of *Ae. albopictus*, whereas vertical transmission rate was 3% and filial infection rate was 0.13% in *Ae. aegypti* controls [67]. This study used mosquito colonies that had been maintained for 9–14 generations in the laboratory, so observations may have been biased by a differential effect of colonization on both species.

Substantial variation among mosquito strains and between DENV strains and serotypes reported in both studies may help to explain conflicting results even when old laboratory colonies were used [69]. Overall, the paucity of solid comparative data prevents firm conclusions on the relative role of *Ae. aegypti* and *Ae. albopictus* in DENV vertical transmission, and its relation with their differential permissiveness to DENV dissemination through oral infection. Additional research is needed to unravel the relationship between rates of virus dissemination and rates of vertical transmission in both mosquito species. In those experiments it will be critical to account for the potential effect of laboratory colonization on vector–virus interactions.

## Conclusions

*Ae. albopictus* will likely continue to spread globally, regardless of efforts to prevent its range expansion. The paucity of historical records of epidemic dengue activity directly associated with *Ae. albopictus,* despite favorable conditions at locations where it was the predominant day-biting *Stegomyia* species, supports the conclusion that *Ae. albopictus* is a less efficient epidemic DENV vector than *Ae. aegypti*. In addition to differences in human blood feeding behavior between the two species, our analysis indicates that lower vectorial capacity is reflected by the lower rates at which *Ae. albopictus* becomes infectious; i.e., lower rates of virus dissemination to salivary glands from the mosquito's midgut. Thus, continued geographic expansion and the replacement of *Ae. aegypti* by *Ae. albopictus* might reduce the risk of epidemic dengue activity. Under most conditions, *Ae. albopictus* would be unlikely to be responsible for large-scale dengue outbreaks. At least for dengue, it is tempting to speculate that the presence of this species constitutes less of a public health threat than *Ae. aegypti*.

The potential role of *Ae. albopictus* in transmission of other arboviruses should remain a concern for public health officials. In the US, for example, areas where La Crosse and eastern equine encephalitis viruses occur must be closely watched. *Ae. albopictus* can potentially act as a bridge vector that brings these viruses into peridomestic environments and, thus, increases risk of human infection. Similarly, *Ae. albopictus* can be an efficient bridge vector for yellow fever and Venezuelan equine encephalitis viruses in Central and South America. This has not been documented to date, despite considerable effort to monitor the possibility. It should be noted that all of these viruses have efficient natural mosquito vectors that maintain them in nature, and we consider it unlikely that the presence of *Ae. albopictus* will change those natural maintenance cycles.

We cannot predict the epidemiological outcome of competitive displacement of *Ae. aegypti* by *Ae. albopictus*. Arboviruses have the potential to rapidly change their host associations, as illustrated by the rapid emergence of epizootic Venezuelan equine encephalitis virus following virus adaptation to an alternative vector through a single amino acid substitution in the envelope glycoprotein [70]. Similarly, recent outbreaks of chikungunya on islands in the Indian Ocean and in Central Africa and Italy indicate that the geographic expansion of *Ae. albopictus* can lead to an increase of this disease. Indeed, laboratory assessments of vector competence associated the recent emergence of chikungunya virus with a single mutation that enhances transmission efficiency by *Ae. albopictus* [71]–[73]. The mutation seems to confer a selective advantage to the virus in locations where *Ae. albopictus* predominates over *Ae. aegypti*, which is typically considered the primary vector of chikungunya virus. Thus, we cannot rule out that displacement of *Ae. aegypti* by *Ae. albopictus* will at some future date be accompanied by virus adaptation to this invasive and increasingly abundant mosquito vector species followed by a global resurgence of chikungunya or other arboviral diseases.

## Methods

### Literature Search

We conducted a thorough literature survey through the ISI Web of Science, NCBI PubMed, and Armed Forces Pest Management Board Literature Retrieval System.

### Meta-analysis

We focused on studies comparing the vector competence of *Ae. albopictus* and *Ae. aegypti* for horizontal DENV transmission based on oral infection (either via membrane or direct feeding). Criteria for inclusion in the meta-analysis were that the studies (i) had directly compared the oral susceptibility of *Ae. albopictus* and *Ae. aegypti* (as opposed to indirectly via a control colony or different replicates), (ii) used mosquitoes from both species that had a similar colonization history (either recently derived from field populations or old laboratory colonies), and (iii) provided sample sizes and raw proportions of infected/uninfected mosquitoes. We only considered “wild-type” viruses and, therefore, excluded studies using attenuated viruses such as vaccine candidates. We also excluded uninformative experiments where all mosquitoes were infected or uninfected. We considered separate experiments from the same study as individual units and assigned a single effect size (i.e., standardized measure of the magnitude of the effect [74]) to each experiment. The analysis was performed on two common measures of vector competence: the proportion of mosquitoes with a midgut infection and the proportion of mosquitoes with an infection disseminated from the midgut to other tissues. The proportion of mosquitoes with a disseminated infection was calculated by including all individuals, including those with an uninfected midgut. We calculated the effect size as the rate difference (RD), which is defined as the difference in rate scores in 2×2 contingency data and ranges from −1 to +1. We arbitrarily assigned negative values to a reduced rate and positive values to a greater rate for *Ae. albopictus* compared to *Ae. aegypti*. When information was available, we noted the serotype, year of isolation, and passage number of virus isolates used. We recorded the duration of the extrinsic incubation period before vector competence was assessed and recorded the number of generations spent by mosquitoes in the laboratory before the experiment was carried out and defined two broad, arbitrary categories: ≤5 and >5 generations of colonization in the laboratory. The cutoff was chosen to distinguish experiments that used mosquitoes during the first few generations after their collection in the field from those that used relatively old colonies that had spent an often-unknown number of generations in the laboratory

All analyses were performed using the software *Metawin 2.0* [74]. The meta-analytic procedure consisted of three steps. First, we calculated effect sizes (RD) and estimated their variances. Second, we assumed no data structure to compile the cumulative effect size of the entire dataset, which is the average effect size weighted by sample size [74]. We also estimated the total heterogeneity (Q_T_) of the dataset and determined its significance against a χ^2^ distribution [74]. Third, we explored the influence of explanatory variables by incorporating data structure in the analysis through one-way models. Importantly, we did not want to assume that there was a common true effect size shared by all experiments. We accounted for the fact that, in addition to sampling error, there was a true random component of variation in effect sizes between experiments by using mixed-effects models that include random variation among experiments and fixed effects of explanatory variables. Mixed-effects models have the advantage of allowing one to generalize results beyond the studies included in the analysis [75]. To test for significance of a variable, total heterogeneity (Q_T_) was partitioned into the variation in effect sizes explained by the model (Q_M_) and the residual error variance in effect sizes not explained by the model. For categorical variables, the difference among groups was determined by testing Q_M_ against a χ^2^ distribution with *n*-1 degrees of freedom (where *n* is the number of groups), whereas for continuous variables, the significance level of Q_M_ was tested against a χ^2^ distribution with one degree of freedom. Because our data set consisted of a relatively small number of experiments, we determined the accuracy of the meta-analytic metrics using bootstrapping procedures and randomization tests [74]. We used simple graphical methods such as examination of weighted histograms of effect sizes, normal quantile plots, and funnel plots [74] to detect any visual indication of publication bias (i.e., the selective publication of articles showing certain types of results over those showing other types of results) in our dataset. We also confirmed the absence of publication bias quantitatively by testing the correlation between the effect size and sample size across experiments using common rank correlation tests, Kendall's *θ* and Spearman's *ρ* [75].

**Key Learning Points**

- Retrospective examination of dengue emergence in the last half century shows that a typical explosive dengue epidemic with hemorrhagic cases has never occurred in places where *Ae. albopictus* predominates over *Ae. aegypti* despite otherwise favorable conditions.
- Experimental assessments of vector competence for dengue viruses indicate that, whereas *Ae. albopictus* is generally more susceptible than *Ae. aegypti* to a midgut infection, *Ae. aegypti* is more competent when virus dissemination to other tissues is considered.
- *Ae. albopictus* susceptibility to dengue virus relative to *Ae. aegypti* tends to increase after a few generations spent in the laboratory, which may have confounded the results of vector competence studies conducted with old laboratory colonies of mosquitoes.
- The paucity of experimental data on the relative ability of *Ae. albopictus* and *Ae. aegypti* to transmit dengue viruses to their offspring, in addition to the potentially confounding effect of mosquito colonization history, prevent firm conclusions on the role on both mosquito species in vertical transmission of dengue viruses in nature.
- *Ae. albopictus* is currently a less efficient vector of dengue viruses than *Ae. aegypti*, but this does not preclude future viral adaptation for enhanced transmission by *Ae. albopictus* in places where this species displaces *Ae. aegypti*.

**Five Key Articles in the Field**

1. Rosen L, Shroyer DA, Tesh RB, Freier JE, Lien JC (1983) Transovarial transmission of dengue viruses by mosquitoes: *Aedes albopictus* and *Aedes aegypti*. Am J Trop Med Hyg 32: 1108-1119.
2. Rosen L, Roseboom LE, Gubler DJ, Lien JC, Chaniotis BN (1985) Comparative susceptibility of mosquito species and strains to oral and parenteral infection with dengue and Japanese encephalitis viruses. Am J Trop Med Hyg 34: 603-615.
3. Vazeille M, Rosen L, Mousson L, Failloux AB (2003) Low oral receptivity for dengue type 2 viruses of *Aedes albopictus* from Southeast Asia compared with that of *Aedes aegypti*. Am J Trop Med Hyg 68: 203-208.
4. Ponlawat A, Harrington LC (2005) Blood feeding patterns of *Aedes aegypti* and *Aedes albopictus* in Thailand. J Med Entomol 42: 844-849.
5. Delatte H, Desvars A, Bouétard A, Bord S, Gimonneau G, et al. (2010) Blood-feeding behavior of *Aedes albopictus*, a vector of chikungunya on La Réunion. Vector Borne Zoonotic Dis 10: 249-258.

## Supporting Information

Table S1 References of studies used in the meta-analysis of relative oral susceptibility to DENV of *Ae. albopictus* and *Ae. aegypti*.
(0.05 MB DOC)
