# Supplementary material for: Consequences of the Expanding Global Distribution of Aedes albopictus for Dengue Virus Transmission
Source: PLoS Negl Trop Dis. 2010 May 25;4(5):e646. doi: 10.1371/journal.pntd.0000646 (PMC2876112; doi:10.1371/journal.pntd.0000646)
Supplement: Table S1 — References of studies used in the meta-analysis of relative oral susceptibility to DENV of Ae. albopictus and Ae. aegypti. (0.05 MB DOC) [file pntd.0000646.s001.doc]

**Supporting Table S1. References of studies used in the meta-analysis of relative oral susceptibility to DENV of *Ae. albopictus* and *Ae. aegypti*.**

| **Publication year** | **Reference** | **Number of experiments** | |
| --- | --- | --- | --- |
|  |  | **Infection** | **Dissemination** |
| 1971 | Whitehead *et al.* Trans R Soc Trop Med Hyg 65(5): 661-667 | 20 | 1 |
| 1979 | Jumali *et al.* Am J Trop Med Hyg 28(4): 717-724 | 0 | 4 |
| 1985 | Rosen *et al.* Am J Trop Med Hyg 34(3): 603-615 | 15 | 0 |
| 1990 | Schoepp *et al.* Am J Trop Med Hyg 42(1): 89-96 | 1 | 0 |
| 1991 | Schoepp *et al.* Am J Trop Med Hyg 45(2): 202-210 | 3 | 0 |
| 1993 | Chen *et al.* J Med Entomol 30(3): 524-530 | 0 | 2 |
| 2001 | Ton Nu *et al.* Ann Soc Entomol Fr 37(4): 473-479 | 0 | 2 |
| 2002 | Johnson *et al.* Am J Trop Med Hyg 67(3): 260-265 | 1 | 1 |
| 2003 | Vazeille *et al.* Am J Trop Med Hyg 68(2): 203-208 | 0 | 21 |
| 2004 | Moncayo *et al.* Emerg Infect Dis 10(10): 1790-1796 | 3 | 3 |
| 2006 | Higgs *et al.* Am J Trop Med Hyg 75(5): 986-993 | 4 | 4 |
| 2007 | Moore *et al.* J Am Mosq Control Assoc 23(4): 383-388 | 2 | 1 |
| 2008 | Alto *et al.* Am J Trop Med Hyg 79(5): 688-695 | 1 | 1 |
| 2009 | Paupy *et al.* Vector Borne Zoonotic Dis (in press) | 0 | 12 |
|  | **Total** | 50 | 52 |

For each study, the number of experiments reporting rates of virus infection and/or dissemination is indicated. Studies are ranked according to their publication year.
